# Usability and potential impact of a web-based literacy-oriented intervention for community-dwelling patients with complex care needs: a mixed methods case report

**DOI:** 10.5195/jmla.2026.1756

**Published:** 2026-01-01

**Authors:** Pierre Pluye, Vera Granikov, Virginie Paquet, Francesca Frati, Fabio Balli, Jiamin Dai, Reem El Sherif, Quan Nha Hong, Roland M. Grad

**Affiliations:** 1 Formerly of the Department of Family Medicine, McGill University, Montréal, Canada; 2 vera.granikov@chairepartenariat.ca, Centre Hospitalier de l'Université de Montréal Research Centre (CRCHUM), Montréal, Canada; 3 virginie.paquet@umontreal.ca, Service des bibliothèques (Recherche et communication savante), Université de Montréal, Montréal, Canada; 4 francesca.frati@mcgill.ca, Schulich Library of Physical Sciences, Life Sciences, and Engineering, McGill University, Montréal, Canada; 5 info@fabioballi.net, Breathing Games Association, Switzerland; 6 jiamin.dai@mail.mcgill.ca, School of Information Studies, McGill University, Montréal, Canada; 7 reem.elsherif@mail.mcgill.ca, Department of Family Medicine, McGill University, Montréal, Canada; 8 quan.nha.hong@umontreal.ca, École de réadaptation de l’Université de Montréal, Université de Montréal, Montréal, Canada; 9 roland.grad@mcgill.ca, Department of Family Medicine, McGill University, Montréal, Canada

**Keywords:** Consumer health information, ehealth literacy, literacy program, online health information

## Abstract

**Background:**

Community-dwelling patients with complex care needs (hereafter “patients”) seek information to choose optimal care. However, patients with low ehealth literacy often have difficulty finding trustworthy, easy-to-understand information. Improving their ehealth literacy can lead to multiple positive health outcomes. This study aimed to describe patients' perceptions of the usability and potential impacts of a web-based, ehealth literacy–oriented intervention.

**Case Description:**

To support patients in finding, appraising, and using online health information (the three core principles of ehealth literacy) we developed the Online Health Information Aid (OHIA), which includes a website, an educational video, and a game. An evaluation was conducted with five patients who received the intervention. Pre-intervention (Day 1) and post-intervention (Day 30) data were collected. Quantitative data were analyzed using descriptive statistics, and qualitative data were analyzed using content analysis. Quantitative and qualitative results were compared in a joint display. Participants included three women and two men aged 46 to 71 years (mean age: 62) with two to 11 chronic health conditions (mean: 5) and two to 20 medications (mean: 10). Participants found the website usable (e.g., “good tool”). For the video, usability scores were high (67%-96%; mean: 79%) with positive comments (e.g., “good and helpful”). However, the game's usability was lower (40%-78%; mean: 60%), and comments were negative (e.g., “complex and not readable”). For three participants, ehealth literacy levels (n=2) and/or knowledge for appraising online health information (n=2) increased post-intervention. However, they did not perceive any impact of the intervention.

**Conclusion:**

These results suggest that the OHIA intervention, specifically the website and the video, is a promising approach to improving ehealth literacy among people with lower education, and a family income below or around the poverty line, including patients with complex care needs.

## BACKGROUND

Patients with complex care needs (PCCN) often navigate fragmented care systems and face additional challenges when trying to access and assess reliable health information online. PCCN living in the community face multiple issues (e.g., multimorbidity, polypharmacy, mental health, social vulnerability) and barriers to optimal use of health and social services (e.g., limited awareness of available options, services, and treatments, paired with low motivation or confidence) [1–4]. Finding, evaluating, and using online health information is another challenge. As is the case for most, many PCCN have difficulty distinguishing trustworthy from misleading health information, and their information needs often remain unmet due to limited time and unclear communication from health professionals [5].

Online health information is generally associated with positive outcomes such as improved health literacy, empowerment, self-care, engagement in healthcare, quality of life, as well as decreased worries [6–12]. Being informed can decrease unnecessary calls and visits to health care professionals and optimize service utilization [12, 13]. Trustworthy information that is easy to read or listen to, for example narrated educational videos, can help reduce health information disparities by addressing gaps in access among marginalized groups [14]. In contrast, misleading information can increase anxiety, deteriorate relationships with health professionals, and cause unnecessary emergency department visits [15–20].

As the proportion of PCCN increases with the aging of the population and the rising prevalence of chronic disease, these individuals continue to face difficult decisions, unmet care needs, fragmented care, inadequate quality of care, and poor health outcomes [21–24]. Moreover, they and their caregivers have greater health information needs to support decision-making about treatment, manage behavioral and psychosocial issues, address concerns about quality of life and autonomy, and navigate the complexities of health and social services [4]. More than half of PCCN have a low level of health literacy across the 18 OECD countries. For example, in Canada, this includes about 60% of PCCN [25]. They face difficulties finding, evaluating, and using online health information that is easy to read, listen to, or watch [26].

Several randomized controlled trials showed that web-based interventions can improve health literacy on specific topics, and health education videos and games can improve the knowledge, attitudes, and behaviors of people with low literacy [27–33]. Videos are more acceptable in a low literacy population and may help reduce informational inequities related to health literacy [34]. Moreover, health education games can improve motivation, engagement, attitude, and learning [35, 36]. However, more research is needed to evaluate the effects of interventions designed to improve ehealth literacy among PCCN with low literacy levels, including the effectiveness of web-based tools such as videos and games for this population.

## CASE PRESENTATION: THE ONLINE HEALTH INFORMATION AID (OHIA) WEBSITE-VIDEO-GAME INTERVENTION

Our team implemented an educational intervention that includes a website called Online Health Information Aid (OHIA) that promotes health literacy skills, accompanied by a video and a game, which aim to promote the use of the website [37]. Overall, this three-component intervention aims to improve ehealth literacy (i.e., skills and confidence for finding, assessing, and using trustworthy health information online). In this case report, we examine the usability of the intervention from the perspective of community-dwelling PCCN, explore the intervention's potential impact, and assess the feasibility of an experimental evaluation.

Our intervention has three components: a website, an educational video, and a game. The purpose of the website is to promote users' (a) ehealth literacy skills for finding, evaluating, and using trustworthy online health information (i.e., knowledge), (b) trust in this information (i.e., attitude), and (c) the use of this information in clinical encounters (i.e., behavior). The website was developed using a user-centered approach and is based on research evidence from a systematic literature review and a qualitative research study [20, 38, 39]. The website provides actionable recommendations and a list of trustworthy sources, in English and in French.

The educational video is a 6-minute animation integrated into the site's homepage, available in English and in French. Its development was informed by international best practice guidelines [40–43] and drew on the Theory of Reasoned Action [44]. This theory proposes that behavior is shaped by a person's knowledge, attitudes, perceived social norms, and sense of control, and it can be applied in studies of ehealth literacy and educational videos. The animated character in the video presents the sections of the OHIA website (i.e., core elements of ehealth literacy) and illustrates how the website can be useful.

The game aims to help users distinguish trustworthy from misleading information. Players are presented with several types of information and are asked to rank them on a scale from 0 (potentially misleading source and content) to 5 (trustworthy source and content). Informed by research evidence, the game uses engaging stories to share knowledge, presents problems for players to solve, and encourages repeated play [35].

### Evaluation

After developing and implementing the OHIA website-video-game intervention, we performed a two-step evaluation to explore its usability and potential impact. These explorations allowed us to assess usability, a critical determinant of impact, as non-usable solutions are unlikely to achieve meaningful outcomes. The study was approved by the McGill University Research Ethics Office.

Through patient organizations and our networks, we recruited five PCCN who met two criteria: (1) a high school education or less, and (2) a family income below or around the poverty line, which is a combination associated with lower levels of ehealth literacy [45]. Although seemingly small, this number of participants is considered sufficient to uncover major flaws and over 80% of usability issues [46–49], as well as to explore a phenomenon and formulate hypotheses [50].

Data collection and analysis were guided by a conceptual framework that describes four levels of information outcomes [39]. Level 1, situational relevance, refers to whether a person finds the information relevant in their specific context. For example, PCCN will continue to read or listen to a webpage if it matches their needs but skip it if not. Level 2, cognitive impact, describes positive or negative cognitive effects of relevant information. For example, PCCN can either learn something new or not understand the information. Relevant information with positive cognitive impact is more likely to be used. Level 3, information use, includes conceptual, legitimating, symbolic, or instrumental uses. For instance, PCCN may use information to decide whether to consult a professional (instrumental) and share it with them (symbolic). However, information use does not necessarily lead to health outcomes. Level 4, health outcomes, refers to positive or negative effects on health and well-being, such as feeling reassured or more anxious after using the information.

### Data Collection

Our evaluation followed a convergent mixed methods design [51]. Quantitative and qualitative data were collected in two steps: Step 1 (Day 1) and Step 2, one month later (Day 30). Our quantitative question was: To what extent can the intervention contribute to improving the level of ehealth literacy? Our qualitative question was: From the participants’ perspective, what are the usability and potential impacts of the intervention? All participants participated in both steps. Each participant received a compensation of $100 Canadian.

On Day 1 (Step 1), we collected baseline quantitative data (see Appendix 1 for the tools and measures used), including sociodemographic information, comorbidities and medication use, ehealth literacy levels, and knowledge to distinguish trustworthy from misleading health information online. Questions related to health literacy were based on the Digital Health Literacy Instrument (DHLI), a validated tool that measures self-reported skills in computer operation, navigation, information searching, evaluating reliability, assessing relevance, creating content, and protecting privacy [52]. Questions assessing knowledge to distinguish trustworthy from misleading information were derived from a systematic literature review on trust and credibility in web-based health information seeking [53]. After the intervention, which involved visiting the website, watching the video, and playing the game, we also collected usability measures. Finally, we gathered qualitative data through semi-structured online interviews, which lasted an average of 82 minutes (range: 63-100 minutes).

On Day 30 (Step 2), to explore the potential impact of the intervention, we collected data about change in ehealth literacy and knowledge to distinguish trustworthy from misleading health information. During the interviews, a research professional asked each participant if the intervention influenced their information searches performed during the last month, if they experienced any benefits for themselves or their caregivers, and perceived risks or negative consequences (e.g., anxiety). The interviewer also asked what participants liked about the intervention and what could be improved. Each interview lasted on average 54 minutes (range: 41-75 minutes).

### Data Analysis

The statistical analysis of quantitative data was descriptive and exploratory. The qualitative content analysis focused on usability of the intervention, its potential to improve ehealth literacy, and the influence of the video and the game on using the website. Interviews were transcribed verbatim and analyzed by two researchers. To compare Steps 1 and 2, quantitative and qualitative results were displayed in a single table, juxtaposing quantitative results on sociodemographic characteristics, contextual factors, ehealth literacy scores, and usability, with qualitative findings, thereby enhancing the interpretation of potential patterns.

## RESULTS

Participants included were three women and two men, French-speaking, aged 46 to 71 years (mean = 62 years), with two to 11 chronic health conditions each (mean = 5), including chronic pain (n=5), diabetes type 2 (n=3) and hypertension (n=2). These problems required two medications per day for two participants, 10 to 15 for two participants, and about 20 for one participant. For three participants, these conditions limited their daily activities. Four participants reported significant problems in the past month such as health problems (n=4), social problems (n=2), and problems with health services (n=2). Three participants were retired and two were unemployed. All five participants were living with a partner. Three participants reported high social support (77-97%), and two reported moderate support (60-63%).

All participants had a computer, Internet access, and a tablet; four had a smartphone. Two participants reported that they use online health information with their family physician and other medical professionals. For example, one of them (P2) described searching for medication-related information and sometimes reading patient forums: “The Internet has been extraordinarily helpful in keeping me informed, talking with my doctors, and being less anxious.” All participants received the three components of the intervention (i.e., OHIA website, video, and game), which they evaluated as detailed below.

### Usability

Qualitative findings and quantitative results are presented in Table 1. All five participants found that the OHIA website and educational video were more usable than the game. Participants described the website as “good”, “comprehensive”, “helpful”, “friendly”, and “pleasant”. The video was described as “excellent”, “good”, “helpful”, with “nice role-playing situations”. Regarding the game, all interviews revealed negative comments: “too complicated”, “too fast”, “incomprehensible”, “unreadable”, “confusing”, and “uninteresting.” The mean usability scores of the video and the game corroborated the interviews. No participant reported intervention-related worries or stress.

**Table 1:** The OHIA intervention: Usability of the three components

Participants	Website usability	Video usability	Score (%)	Game usability	Score (%)
	Interview	Interview		Interview	
P1	“Good tool with tips; quite comprehensive”	“Good; helpful; exemplar”	67	Complicated, too fast, characters too small: “you can't see anything.”	40
P2	“Well done; this will help; this is 95% what I am doing”	“Difficult to find”	82	Complicated, difficult, characters too small: “boxes hard to open.”	64
P3	“Good; with answers to our questions”	“Little slow beginning”	69	Complicated, incomprehensible, uninteresting: “unreadable, too much text.”	44
P4	“Well done and friendly; this helps to understand”	“Great introduction and scenario”	96	Uninteresting: “difficult to see, too many indications” (instructions)	78
P5	“Very well done; easy and agreeable; well explained”	“Excellent, great pictures, well explained, but narration a little bit too slow”	82	Complicated, difficult, uninteresting: “statements too small, confusing game”	76
Mean score	-	-	79.2	-	60.4

Quantitative results are presented in Table 2. For two participants (P1 and P5), the intervention may have improved their ehealth literacy score (+14% and +16% respectively). For two participants (P1 and P3), the intervention may have improved knowledge to distinguish trustworthy from misleading health information (+23% and +41% respectively). These three participants had lower pre-intervention scores, indicating room for improvement.

**Table 2:** Potential impacts of the OHIA intervention (website, video, game)

Participants	ehealth literacy score (%)		
	Pre-intervention	Post-intervention	Difference
P1	71	85	+14
P2	86	81	−5
P3	71	77	+6
P4	80	86	+6
P5	66	82	+16
Participants	Knowledge score (%): capacity to distinguish trustworthy from misleading information sources		
	Pre-intervention	Post-intervention	Difference
P1	52	75	+23
P2	71	67	−4
P3	17	58	+41
P4	96	92	−4
P5	77	79	+2

Comparing quantitative results and qualitative findings revealed valuable insights; divergence was observed in four cases. Three participants improved their scores of ehealth literacy and knowledge for appraising information between the pre- and post-intervention period (Day 1 and 30) but did not qualitatively perceive any impact linked to the intervention (P1, P3 and P5). One participant's score did not improve, but they felt their ability to appraise information had improved after the intervention (P2). In contrast, qualitative findings supported the quantitative results for one participant who neither improved their score nor perceived any impact from the intervention (P4).

## DISCUSSION

Our intervention evaluation results are both encouraging and informative. First, regarding usability, participants unanimously praised the OHIA website and video, suggesting only minor improvements. In contrast, they all found the game difficult to use, highlighting the need for further user-centered design iterations. Second, three participants with lower pre-intervention scores (i.e., ehealth literacy and knowledge to appraise information) improved their scores one month after the intervention. This finding leads to the following hypothesis for future research: in a population with low ehealth literacy, the OHIA website and video can improve ehealth literacy, and knowledge to distinguish trustworthy from misleading health information. The divergence between the quantitative and qualitative results may be attributed to differences between individual perspectives and empirical measurements.

Our results build on existing literature that shows that online health information and web-based literacy-oriented interventions are typically beneficial to patients and caregivers by suggesting that this is the case for PCCN. The OHIA website and video may help patients, health professionals and health information professionals. Patients and caregivers can use the website and video as needed and share this information with their entourage, as demonstrated by two participants (P4, P5). Health professionals can use the OHIA website and video to find information for their patients and encourage their patients to use it. Health information professionals can recommend these resources to their users and incorporate them in educational interventions. The OHIA website and video have been referenced in academic library guides at McGill University and Université de Montréal, which indicates that these resources are accessible to a broad audience, including individuals with higher literacy levels.

Our results show that it is possible to improve ehealth literacy among people with lower education, and a family income below or around the poverty line, including PCCNs. In a growing population of PCCN, even a small improvement can have a meaningful impact. Such gains are important because ehealth literacy constitutes a major determinant of health and is the best predictor of health after smoking, ahead of low income and low education [54–56]. Low ehealth literacy has well-documented negative effects on care, health outcomes, and service use, contributing to higher healthcare utilization, increased costs, and greater health inequities [7, 25, 55, 57–77].

Multiple types of interventions are promising for improving ehealth literacy [33], and the OHIA website and video can contribute. Future research can assess whether the OHIA website and video can help improve ehealth literacy in the general population, and especially how to think critically about the information they encounter. The OHIA website and video can play a particularly important role in the current context of rapidly expanding, targeted, and convincing AI-generated mis- and disinformation, which often spreads with insufficient or no regulatory guardrails [78, 79].

## LIMITATIONS

Our sample may have included ‘ideal’ individuals who are well-positioned to manage their care using trustworthy information that reassures them, as well as individuals who are inclined to resist care [80]. This heterogeneity could have enhanced the potential positive effects of the intervention relative to those that may be observed in a statistically representative sample of PCCN with uniformly low eHealth literacy. Nevertheless, this diversity enabled us to compare participants with lower and higher ehealth literacy, generating valuable insights.

In addition, two key limitations of case reports are the inability to statistically generalize findings and to attribute observed outcomes directly to the intervention. For example, the measured impacts might have resulted from a mere measurement effect or a test-retest effect [81]. Despite these limitations, case reports have merit when they suggest plausible hypotheses that can be tested in future research [82].

## CONCLUSION

In today's context of rapidly advancing generative AI tools, and given the complexity of their needs, it is essential to continue supporting PCCN in acquiring trustworthy evidence-based information, thinking critically, and avoiding misleading content through literacy-oriented programs and educational interventions. The OHIA website and video have the potential to improve ehealth literacy for PCCN and the broader public, and should be promoted through varied media channels, with targeted outreach to health information professionals.

## Data Availability

Due to the small number of participants and participants’ privacy concerns, data could be shared only after the principal investigator's rigorous revision of the justifications and circumstances, and the obtention of a formal agreement of the Research Ethics Office of the Faculty of Medicine and Health Sciences of McGill University, and ultimately the approbation of the participants themselves.

## SUPPLEMENTAL FILES

**Appendix A:** Measurement tools
