# Supplementary material for: Usability and potential impact of a web-based literacy-oriented intervention for community-dwelling patients with complex care needs: a mixed methods case report
Source: J Med Libr Assoc. 2026 Feb 17;114(1):38–45. doi: 10.5195/jmla.2026.1756 (PMC12947933; doi:10.5195/jmla.2026.1756)
Supplement: Supplementary file 1 — Appendix A: Measurement tools [file jmla-114-1-38-s01.pdf]

APPENDIX 1.

Table 1. Assessment Tools and Measures

**Effects (Dependent Variables)**	**Indicators**	**Day 1**	**Day 30**
Use of the OHIA website and related video	Website (based on username): Google Analytics (n visits x n pages/visit x n seconds/page); most visited pages; and average time/page. Video (individual link): play time, scores at each stage, and re-rating score.	No	Yes
Digital Health Literacy	DHLI score: 21 questions with 5 response options per question (validated and tested for reliability) [1].	Yes	Yes
Knowledge to distinguish trustworthy from misleading sources of health information	Score: 54 questions (3 answer options per question) based on a systematic review of the literature [2].	Yes	Yes
Number of online searches for health information	Number of searches per week (critical incident technique and journey mapping technique).	No	Yes
Information use during a meeting with a health professional	Number of past encounters (critical incident technique and journey mapping technique) and future encounters (intention to use information).		
**Independent Variables**	**Indicators**	**Day 1**	**Day 30**
Level of complexity	Perceived level of complexity score from the perspective of people with complex care needs: 38 questions with 5 response options per question (validated questionnaire) [3].	Yes	No
Number of chronic conditions	Items from the CWF 2011 questionnaire on experiences with health and health services (validated in French) - people with more chronic conditions tend to conduct more information searches [4].	Yes	
Number of medications	Items from the CWF 2011 questionnaire on experiences with health and health services (validated		

	in French) - people who take more medication tend to conduct more information searches) [4].		
Priorities	Number of important and urgent issues among 42 social and health issues for people with complex care needs (based on systematic literature review and qualitative research) [5, 6].	Yes	
Age	Number of years (tend to conduct more information searches).	Yes	
Gender	Female, Male, Other or do not wish to answer (women look more frequently for online health information).		
Language	Categories (speakers of multiple languages tend to conduct more information searches to corroborate results).		
Level of education	Categories: High school not completed, or completed, or Grade 13 completed (people with higher levels of education tend to conduct more information searches).		
Annual family income	Categories: Single (0-10K$; 11-20K$*; 21-30K$; >30K$) or non-single (0-20K$; 21-40K$*; 41-60K$; >60K$).		
Level of social support	Score: 6 questions based on the validated F-SozU questionnaire (caregivers can help overcome the limitations of low individual health literacy) [7].	Yes	
Access to Internet	Score based on the 2018 CEFRIO questionnaire including 13 questions [8].	Yes	
**Moderating Variables**	**Indicators**	**Day 1**	**Day 30**
Satisfaction with health services	Score based on the 2011 CFW validated in French [4].	Yes	No
Attitude of health professionals	Score based on the 2011 CFW validated in French [4].		

Access to health services	Score based on the 2011 CFW validated in French [4].		
Communication skills of health professionals	Score (higher > 18 vs. lower < 18) based on 6 validated questions with 3 response options per question, regarding people's perceived competence of professionals in relation to sharing health information found online [9].	Yes	
Quality of life (self-reported)	Score based on a validated quality of life scale including 16 questions with 7 response options per question [10].	Yes	Yes

REFERENCES

1. Van Der Vaart R, Drossaert C. Development of the digital health literacy instrument: Measuring a broad spectrum of health 1.0 and health 2.0 skills. J Med Internet Res. 2017;19(1):e27.

2. Sbaffi L, Rowley J. Trust and credibility in web-based health information: A review and agenda for future research. J Med Internet Res. 2017;19(6):e218.

3. Laerum E, Steine S, Finset A. The patient perspective survey (pps): A new tool to improve consultation outcome and patient involvement in general practice patients with complex health problems: Psychometric testing and development of a final version. 2004;52(2):201-7.

4. Lévesque J-F, Bénigéri M, Labbé A-M. L'expérience de soins des personnes présentant les plus grands besoins: Le québec comparé - résultats de l'enquête internationale sur les politiques de santé du commonwealth fund de 2011. Québec: Commissaire à la santé et au bien-être; 2012. Report No.: 2550637720.

5. Poitras ME, Hudon C, Godbout I, Bujold M, Pluye P, Vaillancourt VT, et al. Decisional needs assessment of patients with complex care needs in primary care. J Eval Clin Pract. 2020;26(2):489-502.

6. Bujold M, Pluye P, Légaré F. Decision-making and related outcomes of patients with complex care needs in primary care settings: A systematic literature review with a case-based qualitative synthesis. BMC Prim Care. 2022;23(1):279.

7. Kliem S, Mößle T, Rehbein F, Hellmann DF, Zenger M, Brähler E. A brief form of the perceived social support questionnaire (f-sozu) was developed, validated, and standardized. 2015;68(5):551-62.

8. CEFRIO - Centre facilitant la recherche et l'innovation dans les organisations. Vieillir à l'ère numérique. NETendances [Internet]. 2018 Accessed; 9(1):[1-19 pp.]. Available from: https://cefrio.qc.ca/fr/enquetes-et-donnees/netendances2018-vieillir-ere-numerique/.
9. Murray E, Lo B, Pollack L, Donelan K, Catania J, White M, et al. The impact of health information on the internet on the physician-patient relationship: Patient perceptions. 2003;163(14):1727-34.
10. Burckhardt CS, Anderson KL. The quality of life scale (qols): Reliability, validity, and utilization. 2003;1(1):1-7.
